# DECLINE OF AN ONLINE COGNITIVE COMPOSITE ASSOCIATED WITH MODIFIABLE LIFESTYLE FACTORS

**DOI:** 10.1093/geroni/igac059.178

**Published:** 2022-12-20

**Authors:** Chao-Yi Wu, Kirsten Wright, Nathan Hantke, Jeffrey Kaye

**Affiliations:** Oregon Health & Science University, Portland, Oregon, United States; Oregon Health & Science University, Portland, Oregon, United States; Oregon Health & Science University, Portland, Oregon, United States; Oregon Health & Science University, Portland, Oregon, United States

## Abstract

Voluntary, self-administered online assessment platforms have gained popularity in monitoring cognition and lifestyle behaviors. This study used the AARP Staying Sharp online brain health assessment platform to estimate the progression of a cognitive composite and correlate it with six modifiable lifestyle behaviors (physical activity, diet, sleep, stress, social activity, and brain engagement) in 147,939 participants (68.6 ± 7.0 years old; 70% female). A cognitive composite was created using attention, immediate and delayed recall, and working memory tasks and lifestyle behaviors were collected by self-report. A lower frequency of exercise (p=0.01), a higher frequency of sitting (p=0.03), a lower frequency of getting in touch with family and friends (p=0.04), and the inability to manage stress (p=0.02) were associated with a decline in the online cognitive composite. A self-administered online assessment platform may be useful in informing lifestyle modification interventions for risk reduction of cognitive impairment and dementia.

